# Neural Correlates of Subliminal Language Processing

**DOI:** 10.1093/cercor/bhu022

**Published:** 2014-02-20

**Authors:** Vadim Axelrod, Moshe Bar, Geraint Rees, Galit Yovel

**Affiliations:** 1 The Gonda Multidisciplinary Brain Research Center, Bar Ilan University, Ramat Gan, Israel; 2 UCL Institute of Cognitive Neuroscience; 3 Wellcome Trust Centre for Neuroimaging, University College London, London, UK; 4 Athinoula A. Martinos Center for Biomedical Imaging, Massachusetts General Hospital and Harvard Medical School, Charlestown, MA, USA; 5 School of Psychological Sciences; 6 Sagol School of Neuroscience, Tel Aviv University, Tel Aviv, Israel

**Keywords:** continuous flash suppression (CFS), decoding subliminal content, fMRI imaging of unconscious processing, multivoxel pattern classification analysis (MVPA), subliminal language processing

## Abstract

Language is a high-level cognitive function, so exploring the neural correlates of unconscious language processing is essential for understanding the limits of unconscious processing in general. The results of several functional magnetic resonance imaging studies have suggested that unconscious lexical and semantic processing is confined to the posterior temporal lobe, without involvement of the frontal lobe—the regions that are indispensable for conscious language processing. However, previous studies employed a similarly designed masked priming paradigm with briefly presented single and contextually unrelated words. It is thus possible, that the stimulation level was insufficiently strong to be detected in the high-level frontal regions. Here, in a high-resolution fMRI and multivariate pattern analysis study we explored the neural correlates of subliminal language processing using a novel paradigm, where written meaningful sentences were suppressed from awareness for extended duration using continuous flash suppression. We found that subjectively and objectively invisible meaningful sentences and unpronounceable nonwords could be discriminated not only in the left posterior superior temporal sulcus (STS), but critically, also in the left middle frontal gyrus. We conclude that frontal lobes play a role in unconscious language processing and that activation of the frontal lobes per se might not be sufficient for achieving conscious awareness.

## Introduction

What are the limits of unconscious language processing? This question has been intensively researched during last 50 years (for reviews see Kouider and Dehaene 2007; Lin and He 2009; Van den Bussche et al. 2009). Though no consensus has been reached, many behavioral experiments show that subliminally presented text can be processed not only at a relatively low orthographic level (e.g., Dehaene et al. 2001; Devlin et al. 2004), but also at a higher semantic level (e.g., Marcel 1983; Jiang et al. 2007; Costello et al. 2009; Sklar et al. 2012; but see Holender 1986; Abrams and Greenwald 2000). Neuroimaging studies show that the visual word form area (VWFA) (Cohen et al. 2000) is involved in unconscious orthographic word processing (e.g., Dehaene et al. 2001; Kouider et al. 2007), whereas unconscious semantic language processing is most consistently observed along the left posterior STS (Devlin et al. 2004; Nakamura et al. 2007; see also Nakamura et al. 2005). Whereas the central role of frontal lobes in various aspects of conscious language processing is unquestionable (for reviews see Vigneau et al. 2006; Price 2012), the role of the frontal lobes in unconscious language processing remains elusive. In particular, only one study reports activations in the inferior frontal gyrus for subliminally presented words (Diaz and McCarthy 2007). However, this study did not implement subjective/objective awareness reports after each trial (Seth et al. 2008) and it is therefore difficult to confidently determine whether the activations indeed reflect unconscious language processing. It is noteworthy that while such limited empirical evidence for unconscious activations in frontal lobes (e.g., Lau and Passingham 2007; van Gaal et al. 2010) is in line with some prominent theoretical models, such as Global Workspace model (Dehaene et al. 1998), it is also possible that subliminal sensory stimulation in the previous studies was too weak to activate the frontal lobes (Haynes 2009). Specifically, the earlier imaging studies used subliminal priming masking paradigm with a brief stimulus exposure, which could have resulted in insufficient brain stimulation. In addition, a more general limitation of previous studies was that none of them measured awareness on each individual trial during the neuroimaging experiment (Seth et al. 2008). Consequently, if on some trials the primes were visible or at least partially visible (Kouider et al. 2010) they would still be considered unaware and therefore their neural correlates may not reflect only unconsciously processed material.

**Figure 1.**
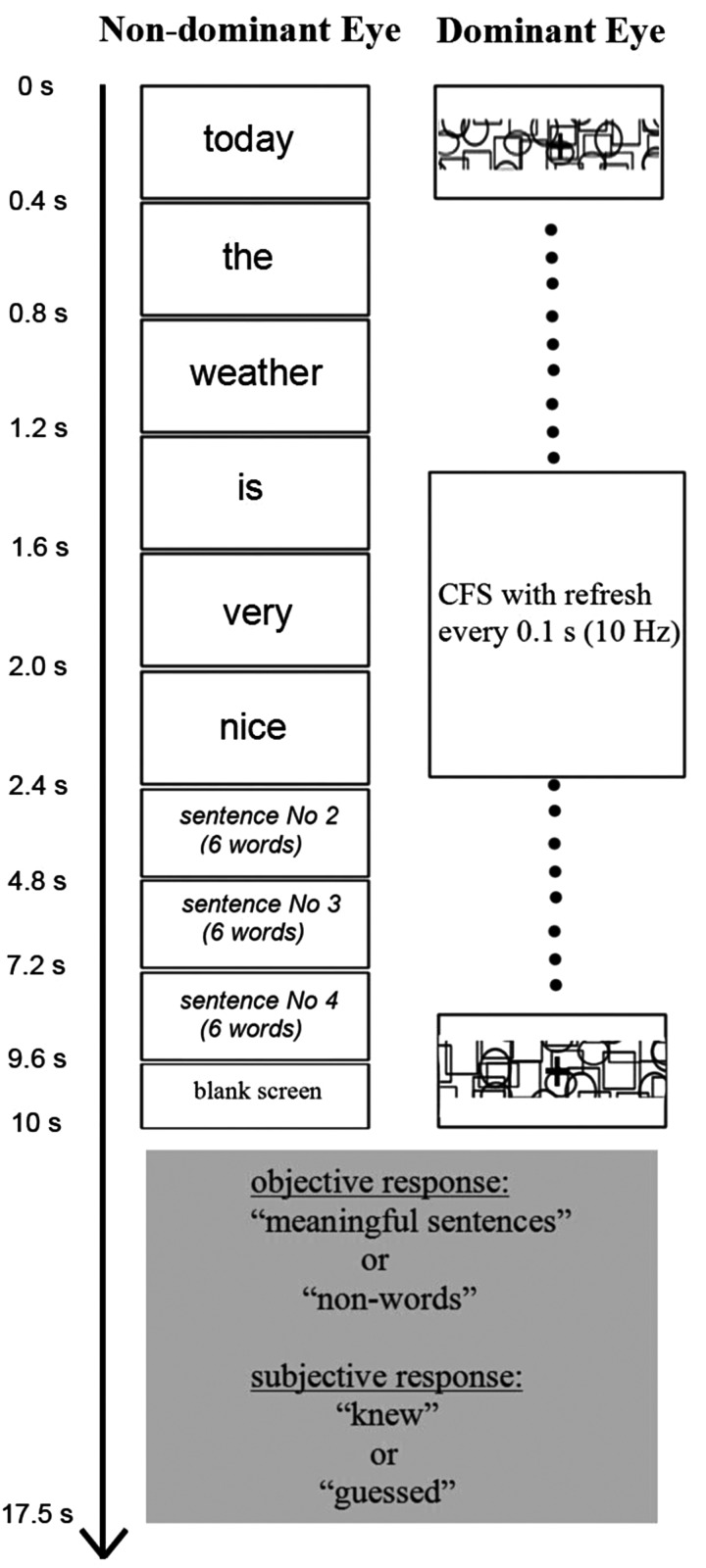
Schematic flow of one block with meaningful sentence in CFS invisible experiment. The words are translated to English for illustrative purpose only while in the experiment all the materials were in Russian. The structure of the blocks with unpronounceable nonwords was the same as the blocks with meaningful sentences, but substituting nonwords (random letter permutations) for words. In the functional localizer (visible experiment) the block flow was similar to that depicted in this figure while the words were visible via both eyes (no CFS mask) and the task was 1-back word repetition (instead of awareness report at the end of the block).

In the current functional magnetic resonance imaging (MRI) study we explored the neural correlates of subliminal language processing, with a novel design that addresses the concerns reviewed above. Observers were presented with series of consecutively presented textual stimuli: meaningful sentences or unpronounceable nonwords (Fedorenko et al. 2010), which were rendered invisible using continuous flash suppression (CFS) (Tsuchiya and Koch 2005) for extended period of time (10 s) (Fig. 1). Critically, as we sought for evidence of unconscious language processing of any type, we decided to use meaningful sentences that required not only semantic, but also syntactic and structural processing—the design which permitted to increase potential differences between meaningful (sentences) and meaningless (nonwords) conditions. After each block of either sentences or nonwords participants reported whether they had been aware of even a single word—a procedure which ensured that data analyses were conducted only on blocks judged invisible by participants. To discriminate between neural activity elicited by the 2 conditions we used multivoxel pattern classification analyses (MVPA) focusing on the language network (Fedorenko et al. 2010), which was localized on a per-participant basis using the same stimuli while they were fully visible. The principal goal of our research was to test whether the frontal lobes were involved in any unconscious processing of language. The secondary goal was to reveal whether using a paradigm that is different from previous studies and by measuring awareness report after each block, the neural correlates of subliminal processing could still be found in the left posterior temporal lobe. Given that different subliminal paradigms do not always yield similar effects (Almeida et al. 2008, 2013; Kanai et al. 2010; Faivre et al. 2012), such a replication is important for establishing this general cognitive phenomenon.

## Materials and Methods

### Apparatus

MRI data were collected using a 3T GE MRI scanner with an 8-channel head coil. Echo planar imaging used a *T*_2_*-weighted sequence to measure changes in blood oxygen level dependent (BOLD) signal. The scanning parameters were as follows: repetition time (TR) = 2.5 s, time echo (TE) = 30 ms, flip angle: 90°, slice thickness: 3.4 mm no gap, field of view (FOV) 200 mm, 32 slices; data were acquired using 96 × 96 matrix (in plane resolution 2.08 × 2.08 mm), reconstruct into 128 × 128 matrix (in plane resolution 1.56 × 1.56 mm). Slice orientation was parallel to temporal lobe with full coverage of the cerebral cortex. An anatomical SPRG scan with full brain coverage was collected with 1 × 1 × 1 mm resolution (TE = 3.52 ms, TR = 9.104 ms).

### Participants

Seventeen healthy volunteers (age: 23–43, 9 females, all right-handed) with normal or corrected-to-normal vision participated in the experiment. The mother-tongue of all participants was Russian (they were born in the Soviet Union and lived there or in an ex-Soviet Union country at least till the age of 14). The study was approved by the ethics committee of the Tel Aviv Sourasky Medical Center. All participants signed informed consent to participate in the study. Data of 2 participants were excluded from the analysis due to inability to follow the instructions (one participant) and excessive movements (>1 cm) in the scanner (another participant).

### Experimental Setup

#### Stimuli

Experiment textual material included series of words presented one word at a time (Fig. 1). The stimuli were of 2 types: meaningful sentences or series of unpronounceable nonwords sentences (random permutation of the letters) (e.g., Fedorenko et al. 2010, 2011). The meaningful sentences described neutral situations (e.g., weather, nature description etc.). The textual material was in Russian (Cyrillic alphabet) written with the Arial font. The letters were presented in lower case (including the first letter of the sentence) and there was no period sign at the end of the sentence. The words were shown at the center of the screen; visual angle size varied between 8 × 5 (horizontal × vertical) and 30 × 5°. The sentences in both conditions were 6 words in length. In total, there were 40 different meaningful and 40 nonword sentences.

#### Continuous Flash Suppression

A standard CFS procedure was used (Tsuchiya and Koch 2005). In the scanner participants wore MR-compatible cardboard anaglyph cyan/red glasses. Stimuli were projected with an LCD projector (NEC, VT660K), positioned ahead of the participant and viewed through a tilted mirror mounted on the MR head coil. Stimuli were projected using the red RGB color channel (visible using red filter) and for the Mondrian mask blue/green RGB channels were used. The red (target) glass filter was always placed over the nondominant eye of each participant. Eye-dominance was assessed prior to the experiment by asking the participants to view a distant object through a hole made by the fingers of their 2 hands (“Miles test”) (Miles 1930; Mendola and Conner 2007). The screen luminance of the text was set to 40% (percent of the maximal screen luminance; dark gray) and of the background was 61% (light gray). The Mondrian pattern was projected on the center of the screen and its size was 34 × 6° of visual angle (to cover the longest word). The pattern of the CFS mask consisted of unfilled ellipses and rectangles, which were similar in image pattern structure to letters (see Fig. 1 for the examples of the pattern). The frequency rate at which the Mondrian patterns were changed was 10 Hz (100 ms for each image).

#### Experimental Design

Participants underwent 2 separate fMRI experiments: the main experiment using CFS masking and a functional localizer with fully visible text. The sessions with visible text were always the last in order not to provide additional cues about the appearance of invisible stimuli. The design and flow of both experiments were identical except for the visibility level of the stimuli and the behavioral task (described below). In the functional localizer experiment, no Mondrian mask was used and the text was projected to both eyes using all 3 RGB channels (to eliminate potential head movements in the scanner between 2 experiments, participants still wore anaglyph glasses during this experiment as well). Screen luminance of the text in the functional localizer experiment was 0% (black) and the background was 61% (light gray).

The experiment used a block-design with each experimental block lasting 10 s and interleaved with a fixation block of 7.5-s duration. Fixation block was an empty screen with “+” sign at the middle (0.2 × 0.2° of visual angle; background luminosity: 61% [light gray color], foreground luminosity: 0% [black color]). Each session consisted of 12 experimental blocks (6 blocks of meaningful sentences and unpronounceable nonwords, respectively). Each session started with a 10 s fixation cross. Total session duration was 3 min and 40 s. Number of sessions per participant varied between participants: for the main experiment (invisibility experiment) it was between 7 and 11 and for the functional localizer experiment it was between 3 and 5. The use of a larger number of short experimental sessions compared with a smaller number of long experimental sessions improves classification performance (Coutanche and Thompson-Schill 2012).

The schematic flow of one block (invisible CFS experiment) consisting of meaningful sentences is shown in Figure 1. The flow of the condition with unpronounceable nonwords was similar. For illustrative purposes only, in the figure the words have been translated to English, while all the experiment materials were in Russian. Duration of a single word was 0.4 s; duration of a single sentence (6 words) was 2.4 s (words appeared one after another without interstimulus interval). Each experimental block consisted of 4 sentences (or 4 lists of nonwords). The sentences appeared back to back without delay between the last word of the previous sentence and the first word of a next sentence. For some sentences of the block the last word of the previous sentence and the first word of the consecutive sentence was identical (e.g., “the queue was extremely long today”, “today the weather is very nice”). The number of repetitions of the last and first word varied randomly between blocks (minimum 0, maximum 2). The first word of each sentence appeared with a random horizontal position jitter (one or 2 letters from centered position). This ensured that when the first and last word of the sentence repeated, there was no effect of word “freezing” on the screen. The blocks of unpronounceable nonwords were similar to blocks of meaningful sentences (Fig. 1) but substituting nonwords (random letter permutations) for words. We decided to use the random letter permutation and not the letter permutation of the real words, since the later can be still occasionally recognized as real words (e.g., Wentura et al. 2005). The 6-word sentence-like structure and the repetition of the last and first word were preserved for nonwords as well.

The behavioral tasks in the functional localizer and in the CFS subliminal experiment were different. In the functional localizer participants were asked to press any button on the response box when they detected a consecutive repetition of a word (1-back task). The repeated words could only be the last and first words of a sentence (see above). This task ensured that participants were attentive to the stimuli.

In the CFS main experiment, participants were required to make 2 separate responses after every block (either 4 sentences or 4 nonword lists, see Fig. 1). These responses were made during the fixation block (no instructions were presented). The participants were first required (“objective response”) whether they thought that a block consisted of sentences (comprising meaningful words) or of nonwords. Participants were then required to make a second response (“subjective response”) indicating whether their first response was based on seeing the stimuli or on a “guess”. Below are the instructions, which were given to participants: “You will be presented with blocks of either sentences composed of meaningful words or series of nonwords. The words or nonwords will be presented sequentially. The stimuli are presented in a way that makes it very hard and probably impossible to see them. At the end of each block you need to make 2 responses. The first response asks you to indicate whether it was a block of sentences (words) or nonwords. Because in each block we present only words or nonwords, detecting one of the stimuli (single word or nonword) during the block would allow you to indicate the correct response. Even if you did not see anything, we ask you to guess. The second response asks you to indicate whether your first response was based on seeing the text or guessing”. It should be noted that as the condition of sentences is comprised of various types of language processing (sentence syntax and structure, words semantics etc.), it was crucial to ensure that any part of language processing remained unconscious. This was the reason that we made it clear to participants, that detecting a single word in the block is sufficient for a correct answer. Participants underwent a short training session outside the scanner as well as a short training session inside the scanner at the beginning of the experiment to ensure that they understood the instructions. Prior to starting the experimental sessions all participants confirmed that the instructions are clear for them. The instructions were also repeated during the experiment, between the sessions. At the end of the experiment, during the informal debriefing, none of the participants indicated any difficulty with performing the task according to the instructions.

### Data Analysis

#### Preprocessing

Data analysis used SPM5 (Wellcome Trust Centre for Neuroimaging, London, UK; http://www.fil.ion.ucl.ac.uk). The first 4 volumes (4 TRs, 10 s) of each session were discarded to allow for T1 equilibration effects. Preprocessing steps applied for functional (EPI) scans included: realignment, slice-time correction, motion correction, normalization to 2 × 2 × 3 voxel resolution using Montreal Neurological Institute (MNI) template and spatial smoothing with a full-width at half-maximum = 6 mm kernel. For the normalization we used a unified segmentation procedure (Ashburner and Friston 2005).

#### Region of Interest Localization

**Figure 2.**
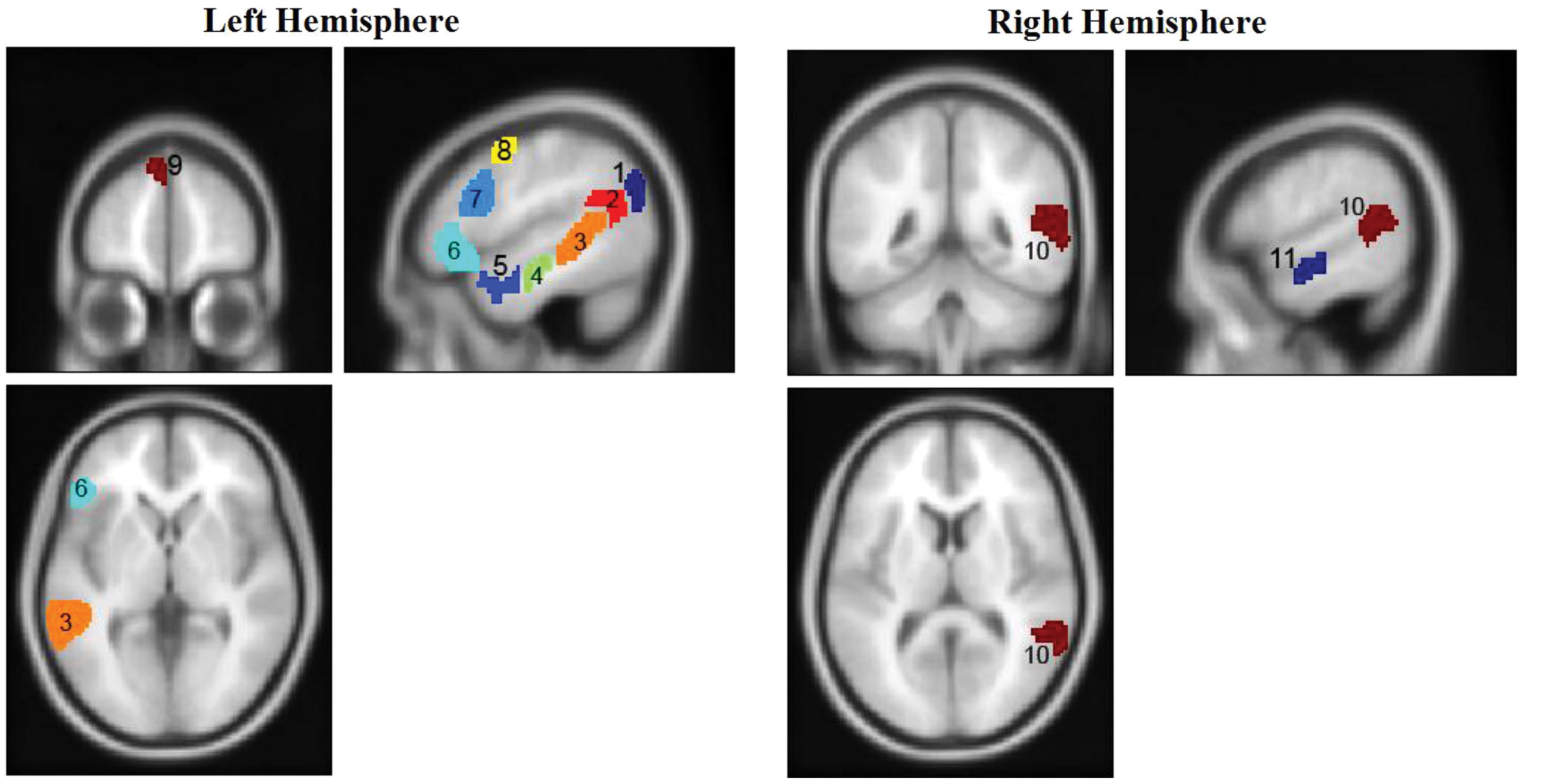
Probabilistic group-level masks of language network (Fedorenko et al. 2010; http://web.mit.edu/evelina9/www/funcloc/funcloc_parcels.html) projected on a SPM template *T*_1_ image. The names of the regions are: 1—left angular gyrus, 2—left supramarginal gyrus, 3—left posterior STS, 4—left middle anterior temporal, 5—left anterior temporal, 6—left orbital inferior frontal gyrus, 7—left inferior frontal gyrus, 8—left middle frontal gyrus, 9—left superior frontal gyrus, 10—right posterior STS, 11—right middle anterior temporal. Regions’ IDs correspond to the IDs in Table 3.

For the language functional localizer (visible text) we estimated a GLM model (HRF boxcar function) with 2 regressors: meaningful sentences and nonwords. We used the contrast “meaningful sentences > nonwords” to identify a network of language processing regions for each participant (Fedorenko et al. 2010, 2011, 2012). To constraint individual GLM-defined functional activations we used probabilistic group-level functional masks (Fedorenko et al. 2010; http://web.mit.edu/evelina9/www/funcloc/funcloc_parcels.html). Thus, for each mask region/participant based on individual “meaningful sentences > nonwords” GLM contrast we selected a contiguous cluster of most selective voxels (number of voxels is specified below). The regions defined by the masks are shown in Figure 2. There were 11 regions in total: 5 regions in the left parieto-temporal lobe (angular gyrus, supramarginal gyrus, posterior STS, middle anterior temporal gyrus, and anterior temporal gyrus), 2 regions in the right hemisphere of the temporal lobe (posterior STS, middle anterior temporal gyrus) and 4 regions in the left hemisphere of the frontal lobe (orbital inferior frontal gyrus, inferior frontal gyrus, middle frontal gyrus, superior frontal gyrus). Critically, as multivariate prediction is influenced by region of interest (ROI) size (e.g., Eger et al. 2008; Walther et al. 2009; Said et al. 2010) we ensured the ROIs of different regions were of an equal size of 100 voxels (1200 mm^3^). In additional analyses we also explored a range of different ROI sizes of 50 and 150 voxels. The ROI size could not be increased further since the size of probabilistic group-level functional masks (Fedorenko et al. 2010) of some of the regions (e.g., left superior frontal gyrus) was <200 voxels. Defining ROIs of equal size was undertaken using custom MATLAB code, where for each region/participant the code selected the contiguous cluster of voxels with the highest *z*-score values relating to the “meaningful sentences > nonwords” contrast in the independent localizer with visible stimuli (similar procedure had been previously applied for face-selective voxels here [Axelrod and Yovel 2012]). The list of the ROIs (100 voxels size) with their coordinates and average *z*-score values can be found in Table 3.

#### Main Experiment: Multivariate Pattern Analysis

A multivariate pattern classification analysis was performed on raw BOLD signal values. After a preprocessing stage (the step which was identical for all EPI data), the data of the main (CFS) experiment were detrended and normalized using the *z*-score MATLAB function. This procedure was applied to the full scan voxel time course. The time course was also shifted 3 TRs to account for hemodynamic lag. In the multivariate analysis the global signal average for each of the 2 conditions was subtracted (e.g., Serences et al. 2009; Misaki et al. 2010), while this procedure was performed separately for each session in order to prevent information leakage in course of cross-validation procedure. Global signal average subtraction, which was applied as part of per-condition normalization procedure, increases classification performance (Raizada et al. 2010; see also Aksoy and Haralick 2001) and may be beneficial, as it prevents voxels with higher values and range to dominate the classifier’'s weights (Coutanche 2013). In addition, subtraction of the global signal average from each condition permits a more straightforward interpretation of the results, as even minimal differences in global average between conditions might be a result of different level of arousal or attention (Coutanche 2013). We obtained qualitatively similar results when the analyses were conducted without subtracting global signal average. The TRs within each block were averaged, resulting in a single average data point value per block that was used as the input to pattern classification analysis. The LibSVM MATLAB implementation of a linear support vector machine was used for classification (http://www.csie.ntu.edu.tw/~cjlin/libsvm/) using a leave-one-session out cross-validation procedure, which was repeated according to number of scans available for each participant. Significance of prediction rate was established using a one-tailed *t*-test above the chance for the group classification rates (Eger et al. 2008; Meyer et al. 2010; Nestor et al. 2011). Multiple comparison Bonferroni correction was made based on the number of ROIs in posterior temporal lobes (7 ROIs, *P*-value significance threshold = 0.0071) and frontal lobe: 4 ROIs, *P*-value significance threshold = 0.0125). Of note, the significance of the results persists when the correction is made based on total number of ROIs (11 ROIs). Supplementary analyses tested whether successful prediction could also be achieved based on signal global level and this analysis differed from the main analysis in 2 ways: 1) the average signal was not subtracted; 2) the timecourses of all voxels in a ROI were averaged resulting in one timecourse (i.e., classification using one dimension). In both main and supplementary analyses only the blocks where the second (“subjective”) report of the participants was “guessed” were used. To ensure that for each participant the equal number of data points (blocks) per condition (otherwise the use of imbalanced data set might bias classification performance [Japkowicz and Stephen 2002]) we randomly discarded the data points from the condition with largest amount of data.

## Results

### Behavioral Results

To establish the level of stimulus awareness (during fMRI scanning) after each block (duration of 10 s) participants were asked to make 2 separate judgments: an objective response (“meaningful sentences”/“nonwords”) and a subjective (confidence) response (“knew”/“guessed”). Most blocks of both conditions were judged to be invisible: the percentage of blocks on which participants responded with “guessed” for meaningful sentences was 80.3% (standard error of mean [SEM] = 4.5%) and for nonwords was 82.6% (SEM = 3.4%). Critically, objective responses for these invisible (“guessed”) blocks were at chance level: meaningful sentences = 51.7% (SEM = 2.1%; *P*-value = 0.22, *t* < 1, one-tailed *t*-test vs. 50%) and nonwords = 51.7% (SEM = 2.4%; *P*-value = 0.24, *t* < 1, one-tailed *t*-test vs. 50%). The distribution of all subjective and objective responses is shown in Tables 1 and 2. In addition, during the informal debriefing after the experiment, we asked the participants what exactly they saw when they choose to answer “guessed”. Critically, all the participants indicated that when they responded “guessed” then they could not see even a single letter within a presented stimulus. Thus, taken together, we conclude that invisibility manipulation was effective and that the trials, which were reported as “guessed”, were genuinely invisible. To explore unconscious processing, we restricted all subsequent analyses to blocks ranked by participants as “guessed” in their subjective response. The average number of blocks per participant/condition was 38.53 (mean squared error [MSE] = 2.49). It was not feasible to investigate neural correlates of conscious processing (“knew” subjective, second response) since there was not sufficient data: 45% of the participants had <5 blocks per condition of this type and the average number of blocks per participant/condition was 7.93 (MSE = 1.94).

**Table 1:** Distribution of “subjective” responses (confidence rating, second response)

	“Knew”	“Guessed”
Meaningful sentences	19.7% (SEM: 4.5%)	80.3% (SEM: 4.5%)
Nonwords	17.4% (SEM: 3.4%)	82.6% (SEM: 3.4%)

**Table 2:** Percent of correct “objective” (first answer) responses per each category (numbers in the table cells) binned for corresponding “subjective” (second answer) response (table columns)

	“Knew”	“Guessed”
Meaningful sentences	62.4% (SEM: 9.9%)	51.7% (SEM: 2.1%)]
Nonwords	86.5% (SEM: 4.9%)	51.7% (SEM: 2.4%)

### Imaging Results

The goal of our analyses was to identify brain regions where pattern signals were sufficient to discriminate subliminal meaningful sentences from nonwords. Our multivoxel pattern classification analysis (MVPA) (Norman et al. 2006) approach focused on nodes in the language network (Fedorenko et al. 2010) identified by an independent localizer using visible stimuli. Summary statistics (average selectivity *z*-score and coordinates) of the ROIs is presented in the Table 3. It can be seen that all the regions except for the left superior frontal gyrus showed higher activation for meaningful sentences compared with nonwords. Notably, the left superior frontal gyrus was also among the less selective regions in the study of Fedorenko et al. (2010). It is noteworthy, that statistical contrast of visible meaningful sentences versus nonwords identifies only the high-level language processing network and does not include inferior temporal cortex (e.g., VWFA [Cohen et al. 2000]), which is implicated in more low-level orthographical processing (Dehaene and Cohen 2011).

**Table 3:** Average *z*-scores and average MNI coordinates (center of mass) of ROI used in the decoding analysis of invisible stimuli

ID	Region of interest	Average *z*-score	MNI coordinates		
			*X*	*Y*	*Z*
1	Left angular gyrus	1.46 (SEM: 0.47)	−43	−73	30
2	Left supramarginal gyrus	3.35 (SEM: 0.64)	−54	−59	15
3	Left posterior superior temporal sulcus (STS)	5.85 (SEM: 0.47)	−57	−42	4
4	Left middle anterior temporal	4.67 (SEM: 0.55)	−57	−18	−9
5	Left anterior temporal	3.9 (SEM: 0.44)	−54	2	−17
6	Left orbital inferior frontal gyrus	3.5 (SEM: 0.51)	−48	28	−3
7	Left inferior frontal gyrus	4.94 (SEM: 0. 64)	−50	17	23
8	Left middle frontal gyrus	3.72 (SEM: 0.62)	−43	1	52
9	Left superior frontal gyrus	0.13 (SEM: 0.47)	−7	54	38
10	Right posterior superior temporal sulcus (STS)	3.24 (SEM: 0.31)	59	−45	8
11	Right middle anterior temporal	3.08 (SEM: 0.47)	54	−14	−13

ID numbers in the first column correspond to the numbers of anatomical masks in Figure 2. The *Z*-score values are based on meaningful sentence > nonwords contrast in visible stimuli localizer. Volume of all ROIs was 100 voxels (1200 mm^3^). Details of how the ROIs were generated are described in the Materials and Methods section.

**Figure 3.**
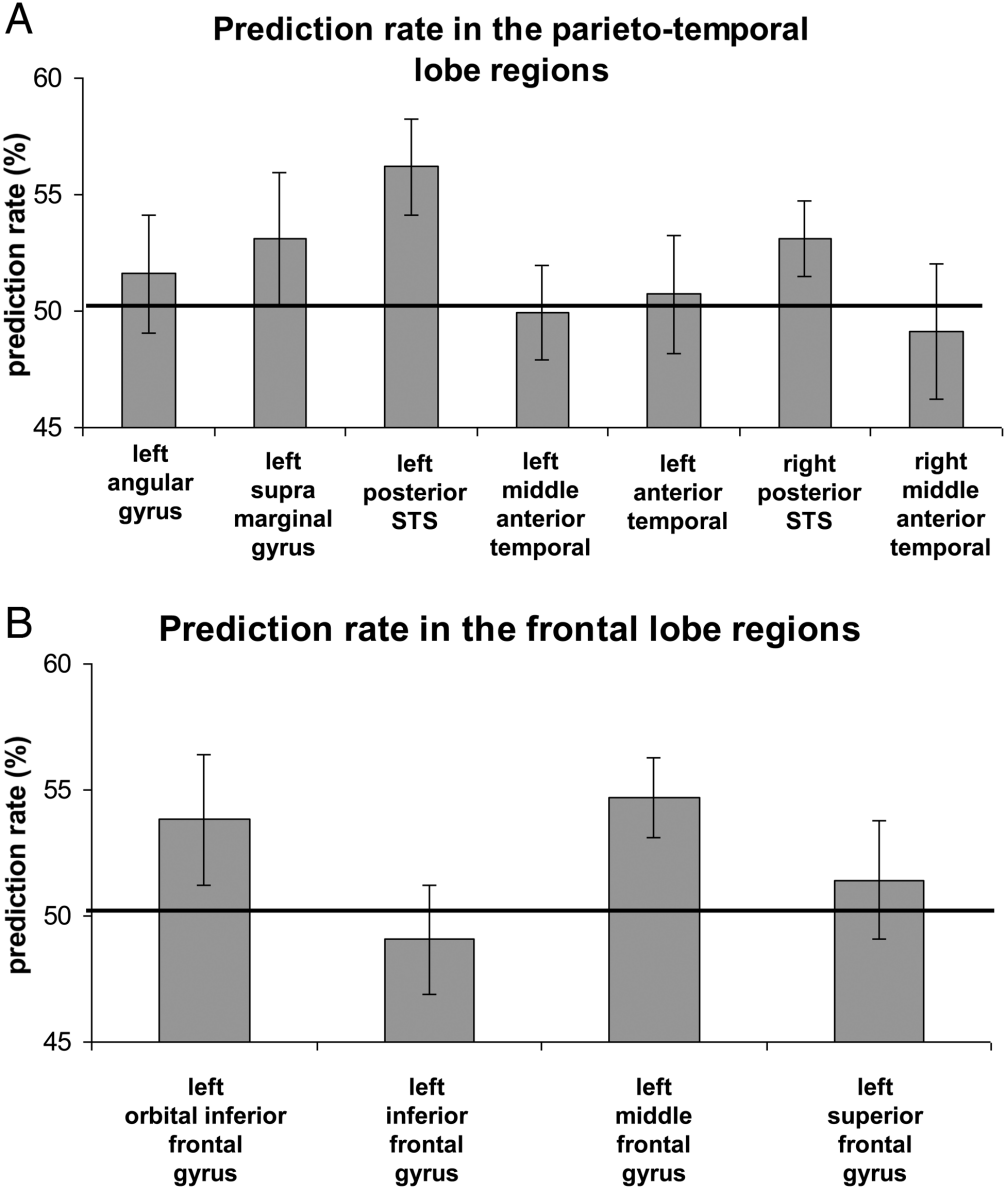
Prediction rate of discrimination between subliminal meaningful sentences and nonwords in language network regions in the temporal lobe (*A*) and the frontal lobe (*B*). Black line is a chance level = 50%; error bars denote standard error of the mean.

The performance of the support vector machine in distinguishing subliminal meaningful sentences from nonwords in the parieto-temporal ROIs is shown in Figure 3*A*. Group-level statistical significance was assessed using one-tailed *t*-tests against chance level of 50% (Bonferroni multiple comparison correction, see Materials and Methods). The only parieto-temporal region, which showed prediction significantly above chance was left posterior STS: 56.2% (MSE: 2%, *t*_(14)_ = 3.01, *P* = 0.004). Prediction rate in the right posterior STS was greater than chance (53.1% [MSE: 1.6%]), but it did not reach statistical significance after multiple comparison correction (*t*_(14)_ = 1.93, *P* = 0.036)]. Performance in the other ROIs did not differ from chance: left supramarginal gyrus: 53.1% (MSE: 2.8%, *t*_(14)_ = 1.1, *P* = 0.22), left angular gyrus: 51.6% (MSE: 2.5%, *t*_(14)_ < 1), left middle anterior temporal: 49.9% (MSE: 2%, *t*_(14)_ < 1), left anterior temporal: 50.7% (MSE: 2.7%, *t*_(14)_ < 1) and right middle anterior temporal: 49.1% (MSE: 2.8%, *t*_(14)_ < 1). To compare the prediction rates between hemispheres, for 2 regions which were localized in both hemispheres (the posterior STS and the middle anterior temporal region) we ran a 2-way repeated measures ANOVA with a region and a hemisphere as factors. The results showed significant main effect of region [*F*_1,14_ = 7.781, *P* = 0.014], but no significant effect of hemisphere [*F*_1,14_ < 1] and no significant interaction [*F*_1,14_ < 1] suggesting that higher prediction rate in the posterior STS comparing to the middle anterior temporal was a property of both hemispheres.

Decoding performance comparing subliminal meaningful sentences and nonwords in frontal regions is shown in Figure 3*B*. The only region which showed prediction rate significantly above chance was left middle frontal gyrus: 54.7% (MSE: 1.5%, *t*_(14)_ = 2.98, *P* < 0.004). In 3 other regions the prediction rate did not differ from chance level: left orbital inferior frontal gyrus: 53.8% (MSE: 2.6%, *t*_(14)_ = 1.47, *P* = 0.081), left inferior frontal gyrus: 49.1% (MSE: 2.2%, *t*_(14)_ < 1), left superior frontal gyrus: 51.4% (MSE: 2.3%, *t*_(14)_ < 1]. To test whether the amount of unconscious information differed between the highest classification rate region in the temporal and frontal lobes we compared the prediction rates in the left posterior STS and left middle frontal gyrus. No significant difference was found (paired *t*-test, *t*_(14)_ < 1), suggesting that there is no evidence that one of the regions contained more information than the other.

So far we have shown that it was possible to discriminate between unconscious meaningful sentences and nonwords based on multidimensional patterns of BOLD signals in the left posterior STS and left middle frontal gyrus. Now, we asked whether the 2 subliminal conditions could also be discriminated based on global signal level alone—the univariate approach, which is extensively used in fMRI research. We therefore conducted additional analyses where the classification was done for only one dimension, which was the average across all the voxels in the ROI. This analysis revealed that across both parieto-temporal and frontal lobe regions only the angular gyrus exhibited above chance prediction rate (53.7%, MSE: 1.8%), but it did not reach significance level after multiple comparison correction [*t*_(14)_ = 2.08, *P* = 0.028]. In all other regions the prediction rate did not exceed 51.5% and did not differ from chance [*t*_(14)_ < 1]. We conclude that average signal did not contain sufficient information for successful discrimination between 2 conditions.

**Figure 4.**
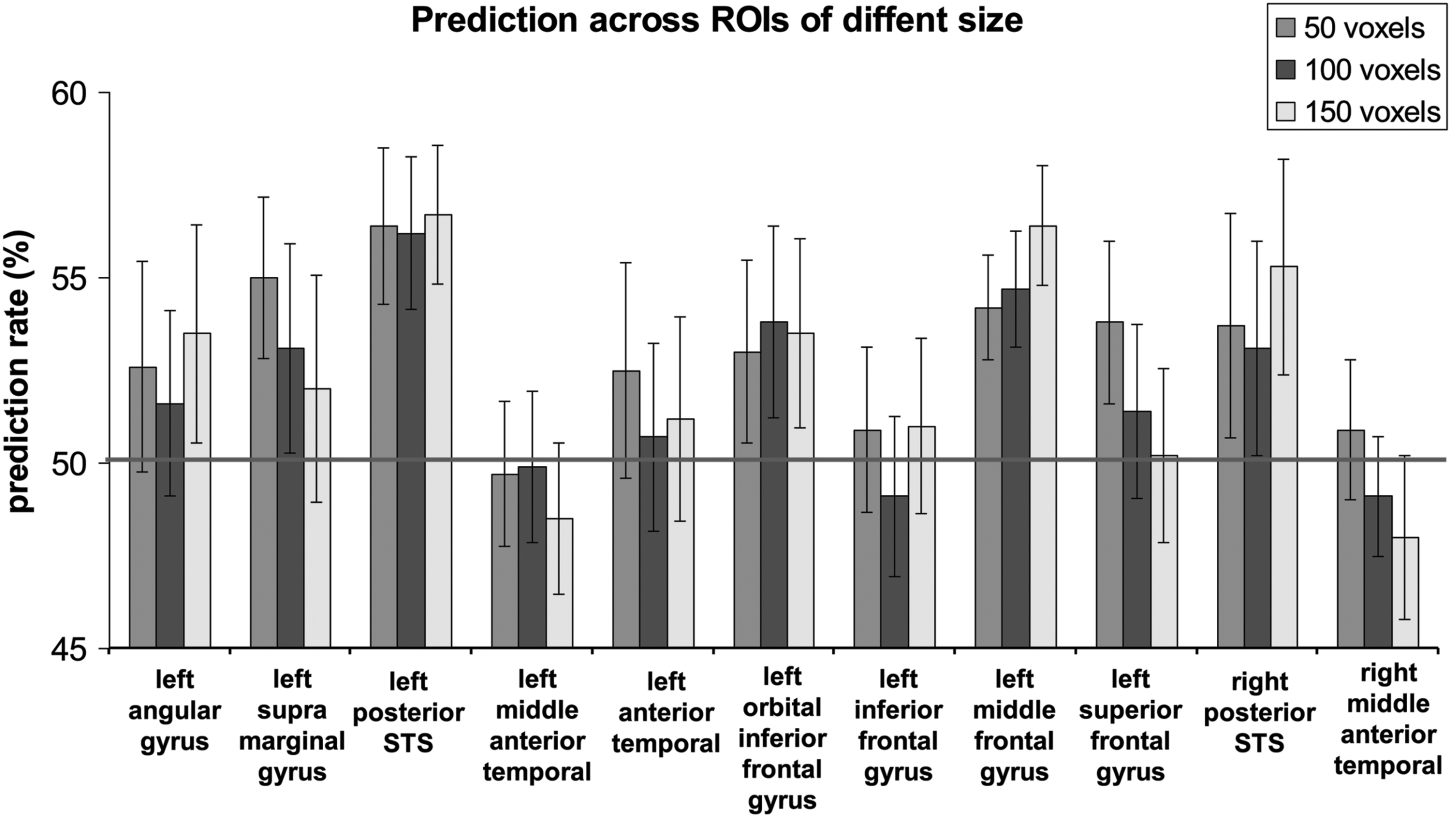
Discrimination between subliminal meaningful sentences and nonwords in language network regions for ROI size of 50, 100, and 150 voxels. Grey line represents a chance level of performance (50%); error bars denote standard error of the mean.

Finally, to ensure that the reported result was not idiosyncratic for a specific ROI size, we repeated the multivariate analyses for the ROI size of 50 and 150 voxels. As in the main analysis, the significance was assessed based on Bonferroni multiple comparison correction for each ROI size (see Materials and Methods). The results of this analysis are shown in Figure 4. Critically, for both left posterior STS and the left middle frontal gyrus the prediction rate was always significantly above chance: left posterior STS [50 voxels: prediction rate: 56.4%, MSE: 2.1%, *t*_(14)_ = 3.02, *P* = 0.004; 150 voxels: prediction rate: 56.7%, MSE: 1.9%, *t*_(14)_ = 3.6, *P* = 0.001)], left middle frontal gyrus [50 voxels: prediction rate: 54.2%, MSE: 1.4%, *t*_(14)_ = 2.99, *P* = 0.004; 150 voxels: prediction rate: 56.4%, MSE: 1.6%, *t*_(14)_ = 3.97, *P* < 0.001]. Consistent with our main analysis the prediction rate was also above chance in the right posterior STS, but statistical significance was not reached after multiple comparison correction [50 voxels: prediction rate: 53.7%, MSE: 1.9%, *t*_(14)_ = 1.94, *P* = 0.04; 150 voxels: prediction rate: 55.3%, MSE: 2.2%, *t*_(14)_ = 2.4, *P* = 0.01]. Performance did not differ from chance in left angular gyrus: [50 voxels: prediction rate: 52.6%, MSE: 2.8%, *t*_(14)_ < 1; 150 voxels: prediction rate: 53.5%, MSE: 2.9%, *t*_(14)_ = 1.19, *P* < 0.12], left supramarginal gyrus: [50 voxels: prediction rate: 55%, MSE: 2.2%, *t*_(14)_ = 2.29, *P* = 0.018; 150 voxels: prediction rate: 52%, MSE: 3.1%, *t*_(14)_ < 1], left orbital inferior frontal gyrus [50 voxels: prediction rate: 53%, MSE: 2.5%, *t*_(14)_ = 1.23, *P* = 0.12; 150 voxels: prediction rate: 53.5%, MSE: 2.6%, *t*_(14)_ = 1.37, *P* = 0.09], left superior frontal gyrus [50 voxels: prediction rate: 53.8%, MSE: 2.2%, *t*_(14)_ = 1.73, *P* = 0.05; 150 voxels: prediction rate: 50.2%, MSE: 2.5%, *t*_(14)_ < 1]. In left middle anterior temporal, left anterior temporal, left inferior frontal gyrus and right middle anterior temporal the prediction rate also did not differ from chance and was <52.5% [*t*_(14)_ < 1]. The results of this analysis suggest that both the left posterior STS and the left middle frontal gyrus contained the information, which permitted reliable discrimination between meaningful sentences and nonwords across different ROI sizes.

## Discussion

The principal finding of this study was that subliminal meaningful sentences and nonwords could be discriminated above chance level based on BOLD signals in 2 regions: the left posterior STS and the middle frontal gyrus. This supports the idea that high-level language information can be processed in the absence of awareness and critically provides important evidence that unconscious language processing is not confined to occipito-temporal lobes, but also involves the human frontal lobes.

The language processing system is a widely distributed brain network, which spans large regions of the left temporal and frontal lobes (for reviews see Vigneau et al. 2006; Price 2012) as well as regions in the right temporal lobe (for reviews see Jung-Beeman 2005; Vigneau et al. 2011). Strong activation of the language network was observed in our functional localizer experiment with visible stimuli, where all the regions except for the superior frontal gyrus showed higher activation to meaningful sentences compared with unpronounceable nonwords (Table 3). Notably, the level of activations and the amount of information is drastically attenuated with unconscious stimulation. Even more relevant for the current discussion, is that some regions like frontal lobes are usually not activated at all during unconscious processing (for review see Dehaene and Changeux 2011)—evidence that is taken to support theoretical models, such as Global Workspace Theory (Dehaene et al. 1998). Yet, alternatively, it can be suggested that weak sensory stimulation, which is an inevitable consequence of rendering stimuli invisible, is simply not sufficiently strong to lead to activation in areas processing high-level information such as the frontal lobes (Haynes 2009). In the current study, to increase the probability of detecting the signals in the frontal lobes we: 1) increased sensory stimulation by projecting invisible stimuli for an extended period of time (10 s); 2) enhanced linguistic processing by showing meaningful sentences, which in addition to semantics also contained syntax and structure; 3) used multivariate ROI analysis approaches that focused on language-selective regions, which were localized using independent experiment with visible stimuli. Critically, by implementing the awareness report procedure after each block we ensured that the only blocks used in our analysis were subjectively and objectively invisible on a per-participant and per-block basis. We found that invisible meaningful sentences and unpronounceable nonwords could be discriminated beyond chance level in the left middle frontal gyrus (Fig. 3*B*). The classification result was stable and did not depend on the exact size of the ROI used (Fig. 4).

It is noteworthy, that because none of the previous studies that have examined unconscious language processing could reliably demonstrate unconscious language activity in the frontal lobes, we deliberately designed the study to maximize the difference between 2 conditions (meaningful sentences vs. nonwords). Accordingly, the current design was not intended to answer the question what type of language information (semantic, syntactic, structural, and semantic context etc.) contributed to successful unconscious discrimination. Yet, the fact that in the conscious language experiments the left middle frontal gyrus has been shown to be most active in studies with sentences (Bottini et al. 1994; Baumgaertner et al. 2002), text processing (Vingerhoets et al. 2003), and complex language material in general (for review see Vigneau et al. 2006) makes it plausible that subliminal syntactic/structural information might have contributed to successful discrimination between meaningful sentences and nonwords. This interpretation is also in line with a recent event-related potentials (ERP) study (Batterink and Neville 2013), where using auditory–visual attentional blink paradigm (Raymond et al. 1992) the authors showed that consciously undetected violations in written sentence syntax processing elicited early frontal negativity ∼100–400 ms. Interestingly, we found no successful decoding in the orbital inferior frontal gyrus and inferior frontal gyrus regions (including Broca's area)—the key regions of language processing in general (Broca 1861). This result is apparently at odds with the results of Diaz and McCarthy (2007) study, who did report extended activations in the left inferior frontal gyrus during a subliminal semantic task. Yet, as this study did not assess awareness after each trial, it is possible that invisibility was not complete and the participants were aware of the subliminal stimuli during some of the trials.

From a broader conscious awareness theoretical perspective, the fact that unconscious information was successfully decoded from a region in the frontal lobes suggests that information can be processed by frontal lobes without automatically triggering conscious awareness. This result is consistent with first order theories (Block 2005) and recurrent processing view (Lamme 2006; van Gaal and Lamme 2011) which do not attribute special role to the frontal lobes in achieving conscious awareness. The present result does not support the original formulation of Global Workspace Theory (Dehaene et al. 1998), which does not expect the frontal lobes to be activated by unconscious stimulation. Yet, based on the recent elaboration of this theory (Dehaene and Changeux 2011), our result might not contradict it either since the activity we report was localized in a specific region and did not span large portions of the frontal lobe, as is the case for many conscious experiences (Dehaene and Changeux 2011). Indeed, localized fMRI activations of the frontal lobes were previously shown in unconscious cognitive control tasks (Lau and Passingham 2007; van Gaal et al. 2008; see also EEG findings: van Gaal et al. 2010, 2011). Yet, the neural systems responsible for cognitive control and language processing are very different. Thus, the present findings complement current knowledge by showing that unconscious language processing can also elicit localized activity in the frontal lobes.

Interestingly, many previous studies using various stimuli, such as faces (e.g., Sterzer et al. 2008; Schurger et al. 2010; Fahrenfort et al. 2012) or words (Devlin et al. 2004; Nakamura et al. 2005, 2007) failed to find unconscious information in the frontal lobes. In the present study, we used a combination of experimental design (long stimulation duration using CFS and meaningful sentences) and data analysis (MVPA) procedures, which has not been previously applied together. There was no way to estimate a contribution of each one of these procedures, as our experiment did not include direct, within experiment comparison, between different parameters (e.g., long stimulation duration using CFS vs. short backward masking stimulation). Having said that, we were able to establish that the use of multivariate analysis (MVPA) played an important role in decoding unconscious activity in the frontal lobes, as the successful decoding could not be achieved using univariate analysis. Similar observation was made by another study, where invisible face/scene stimuli could be decoded in the temporal lobe only by using multivariate, but not using univariate approach (Sterzer et al. 2008). Thus, future studies are needed to examine whether the use of MVPA will permit to find unconscious information in the frontal lobes, also for the backward masking paradigms (Devlin et al. 2004; Nakamura et al. 2005, 2007).

Additional finding of the current study was that subliminal meaningful sentences and nonwords could be discriminated beyond chance level from signals in the left posterior STS. This result is consistent with previous studies that also reported signals associated with unconscious processing of words in this area (Devlin et al. 2004; Nakamura et al. 2005, 2007). Critically, only the present study implemented awareness report after each block allowing characterization of subjective and objective invisibility. Compared with previous studies we used a different methodological approach: experimental paradigm (CFS vs. backward/forward mask priming), language stimuli (sentences vs. words) and data analysis approach (MVPA) (Norman et al. 2006) vs. fMR-adaptation (Grill-Spector et al. 1999)]. Importantly, despite the differences between the studies the result in the left posterior STS was successfully replicated, providing converging evidence and making a strong case for the involvement of the left posterior STS in subliminal text processing. It should be also noted, that discrimination rate from signals in the right posterior STS was also relatively high (Fig. 3*B*) and stable across ROIs of different sizes (Fig. 4), thought it did not reach statistical significance after correction for multiple comparisons. An important role of bilateral posterior STS in semantic processing with visible stimuli has been shown using various tasks and paradigms (for reviews see Jung-Beeman 2005; Vigneau et al. 2006, 2011). As this region was also shown to be involved in syntactic processing (e.g., Ben-Shachar et al. 2004) it remains to be established what type of information contributed the most to successful discrimination of the invisible stimuli. Interestingly, in our functional localizer task with visible stimuli, while the activation of the left posterior STS was the strongest among all the regions (Table 3, average *z*-score column), the activation of the right posterior STS was weaker than most other regions. Thus, successful discrimination of invisible stimuli was not just a direct consequence of a strong level of activation for visible stimuli (Smith et al. 2011; Tong et al. 2012), but might also reflect regional specialization in the absence of aware processing.

Interestingly, while fMRI studies including the current (Devlin et al. 2004; Nakamura et al. 2005, 2007; Diaz and McCarthy 2007) consistently find neural correlates of subliminal meaningful text processing in the left posterior temporal lobe, one recent ERP study, which also used CFS (Tsuchiya and Koch 2005) failed to find any modulation of the N400 semantic component to invisible text content (Kang et al. 2011; see also: Vogel et al. 1998; Heyman and Moors 2012). Though differences in the signal measured by the 2 neuroimaging methods can potentially explain these different results, based on our current findings we propose an additional interpretation. The N400 is thought to originate from multiple sources in the left temporal lobe (Kutas and Federmeier 2011). In the present study we identified 5 regions in the left temporal lobe (Fig. 2) that were all selective to meaningful text processing when stimuli were visible (Table 3). Yet, when the text was presented subliminally out of those 5 temporal regions only one of them (left posterior STS) afforded successful discrimination of meaningful sentences from nonwords, while in the other temporal ROIs performance did not differ from chance level. Given that the N400 ERP component reflects these multiple sources (Kutas and Federmeier 2011), it is possible that the neural activity that originated in discriminative left posterior STS was intermixed with the activity which originated in neighboring, nondiscriminative regions (e.g., left middle anterior temporal, left anterior temporal). As a result, the N400 component recorded on the scalp was not sensitive enough to reflect the subliminal semantic processing.

Finally, a novel methodological aspect of our work was the presentation of long invisible sentences. While the CFS paradigm was previously used for presenting subliminal words (Costello et al. 2009; Kang et al. 2011; Yang and Yeh 2011) or 3 words sentence on one screen (Sklar et al. 2012) here we propose a method of presenting invisible sentences of the unlimited length. The presentation of invisible sentences for prolonged duration (e.g., 10 s in our case) gives an opportunity to present not only subliminal text with much richer semantic information, but also permits exploration of unconscious neural correlates of complex language processing (e.g., syntax processing). We suggest that this paradigm can be useful for future studies in the field.

In conclusion, in the current study we demonstrated that based on the activity in the human left posterior STS and left middle frontal gyrus it was possible to discriminate between subliminally presented meaningful sentences and nonwords. This result supports the notion that high-level language functions might be processed subliminally and provides important evidence that frontal regions might be involved in unconscious language processing.

## Funding

This study was supported by the Israeli Center of Research Excellence in Cognitive Sciences (V.A., M.B., and G.Y.), Daniel Turnberg Travel Fellowship (V.A.) and the Wellcome Trust (G.R.). Funding to pay the Open Access publication charges for this article was provided by Wellcome Trust grant 100227/Z/12/Z and Wellcome Trust strategic award 091593/Z/10/Z.
